# Digital Twins for Multiple Sclerosis

**DOI:** 10.3389/fimmu.2021.669811

**Published:** 2021-05-03

**Authors:** Isabel Voigt, Hernan Inojosa, Anja Dillenseger, Rocco Haase, Katja Akgün, Tjalf Ziemssen

**Affiliations:** Center of Clinical Neuroscience, Department of Neurology, University Hospital Carl Gustav Carus, Technical University of Dresden, Dresden, Germany

**Keywords:** multiple sclerosis, precision medicine, personalized medicine, digital twin, decision analysis, medical care

## Abstract

An individualized innovative disease management is of great importance for people with multiple sclerosis (pwMS) to cope with the complexity of this chronic, multidimensional disease. However, an individual state of the art strategy, with precise adjustment to the patient’s characteristics, is still far from being part of the everyday care of pwMS. The development of digital twins could decisively advance the necessary implementation of an individualized innovative management of MS. Through artificial intelligence-based analysis of several disease parameters – including clinical and para-clinical outcomes, multi-omics, biomarkers, patient-related data, information about the patient’s life circumstances and plans, and medical procedures – a digital twin paired to the patient’s characteristic can be created, enabling healthcare professionals to handle large amounts of patient data. This can contribute to a more personalized and effective care by integrating data from multiple sources in a standardized manner, implementing individualized clinical pathways, supporting physician-patient communication and facilitating a shared decision-making. With a clear display of pre-analyzed patient data on a dashboard, patient participation and individualized clinical decisions as well as the prediction of disease progression and treatment simulation could become possible. In this review, we focus on the advantages, challenges and practical aspects of digital twins in the management of MS. We discuss the use of digital twins for MS as a revolutionary tool to improve diagnosis, monitoring and therapy refining patients’ well-being, saving economic costs, and enabling prevention of disease progression. Digital twins will help make precision medicine and patient-centered care a reality in everyday life.

## Introduction

The technology of digital twins (DTs) is a promising concept that has become the focus of interest in industry and, in recent years, in healthcare sector as well. DTs are a revolutionary tool in phenotyping patients, where analysis of large amounts of data (big data) through new technologies like artificial intelligence (AI) enables visualization of a virtual copy (twin) of the patient at different stages of the disease and supports further therapeutic decisions. However, the use of DTs in medical care and especially in the management of patients is still in its infancy. DTs have enormous potential, especially when it comes to precision medicine: they can be used to simulate individual therapies in advance and visualize potential therapy results and disease progression. The concept of DTs seems to be particularly suitable for the treatment of multiple sclerosis (MS), because this chronic autoimmune “disease of a thousand faces” is characterized by heterogeneous course, complexity and multidimensionality, an increasing number of treatment options and a resulting wealth of data. DTs can significantly improve precision medicine for people with MS (pwMS) by enabling healthcare professionals (HCPs) to handle big data and provide more personalized and effective care. In this paper, we focus on our vision of how to design a DT for the management of MS. The advantages of DTs for the personalized treatment of individual pwMS are highlighted without ignoring the challenges on its development. With our review, we want to answer the question whether “Digital Twins for Multiple Sclerosis” (DTMS) may serve as a game changer in the management of MS.

## Multiple Sclerosis Requires Precision Medicine

### Multiple Sclerosis as a Chronic Multidimensional Disease

MS is a chronic autoimmune, degenerative and lifelong disease of the central nervous system (CNS) and the most common cause of neurological disability in young adults. At a pathological level, the infiltration of immune cells into the CNS manifests as localized demyelinating lesions in the white and gray matters of the brain and spinal cord, observed in pathological specimens as well as in magnetic resonance imaging (MRI) sequences (1). In addition, the disease leads to a progressive destruction of myelin layers (demyelination) and progressive axonal injury, loss and neurodegeneration, impairing the function of the CNS in several ways (2, 3).

MS has different clinical disease courses that have been classically described. Around 85-90% of the patients are diagnosed with a relapsing remitting form of the disease (RRMS) at the beginning (4, 5). These patients are affected by attacks of unpredictable clinical relapses caused by inflammatory demyelinating lesions in the CNS, resulting in a complete or partial recovery of the neurological symptoms. After several years, the majority of these patients if untreated will develop secondary progressive MS (SPMS), where the neurological function decreases over time independent of relapse activity (6, 7). About 10-15% of the patients do not have relapses during the course of the disease. In these patients, the disease already begins with a gradual increase in neurological symptoms. This is called primary progressive progression (PPMS). Often a spastic gait disorder develops over the years, more rarely a progressive cerebellar syndrome (8). Beyond this raw classification of disease courses, each MS patient presents with a very individual course of his MS.

**Longitudinal course**. As described, MS is characterized by a chronic and/or episodic course. PwMS require long-term phenotyping, monitoring and most often treatment with disease-modifying therapies (DMTs) (9). In the early stages of MS, the damage occurring in the brain can still be compensated by the so-called neurological reserve. This compensatory mechanism explains why, on the one hand, early-stage MS is often not diagnosed promptly and, on the other hand, is often not taken seriously enough, especially with regard to negative long-term consequences (10–12). As the disease progresses, the neurological reserve decreases, especially if MS activity is not adequately treated (12). Since therapy started early in the course of the disease has an inhibitory effect on the progression of MS, it should be diagnosed and treated without any delays (13, 14).

**Heterogeneous course and different dimensions**. MS is popularly known as the “disease of a thousand faces” because MS lesions and other abnormalities can occur in the whole CNS usually leading to a variety of neurological deficits including fatigue, visual and bladder problems, pain, spasticity, reduced mobility and sexuality as well as psychological conditions such as depression (15–17). Due to this heterogeneity and the intra-individual unpredictable and inter-individually quite variable course, the diagnosis, phenotyping and monitoring of MS is very challenging (18, 19). The multidimensional disease characteristics of each patient should be made quantifiable to allow phenotyping of the individual disease characteristics and long-term monitoring of these parameters (20). This leads to a large amount of multidimensional data.

**Multidimensional data**. When quantifying MS, it is necessary to distinguish between different dimensions and perspectives. Starting from neurological-clinical parameters, they range from quantitative assessment of individual neurological functional systems (e.g. cognition, gait analysis), through imaging (MRI, ocular coherence tomography (OCT)), electrophysiological methods and the inclusion of patient-reported outcomes (PRO), up to new molecular and digital biomarkers (20). This data can be obtained in the setting of clinical trials or in real world practice, which represents also differences in its collection, volume, veracity and availability. To do justice to the complexity of MS, these parameters must be integrated into detailed individual patient charts as well as into large databases in order to be able to analyze them meaningfully.

**Increasing number of potential therapeutic interventions**. The number of treatment options that intervene in the immune system on different levels can modify disease is increasing (19, 21–25). This growing availability of DMTs is broadening the treatment options towards a more individualized therapy (24). Different mechanisms of action and intervention strategies are linked to individual treatments (26–29). On treatment, the monitoring of MS disease activity is key to achieve optimal outcomes in order to initiate a therapy change or escalation in time in case of an insufficient response (10, 30).

Therefore, the chronic, heterogenic and multifocal “disease of a thousand faces” requires a complex, ubiquitous and differentiated as well as adaptive diagnosis, monitoring and treatment strategy. This strategy should be personalized and tailored to the individual needs and disease course of the patient and be continuously adjusted (25).

### Precision Medicine for People With Multiple Sclerosis

An emerging approach towards personalized treatment is precision medicine, or, as an older term, personalized medicine (31–35), that takes into account individual variability in genes, environment, and lifestyle for each person (32, 36–42). Precision medicine covers diagnosis, treatment and management to achieve better patient outcomes (43). Through precision medicine, it is possible to break down the complexity of the disease. The patterns and inter-individual variability can be better understood. Thereby, precision medicine presents a framework for developing targeted treatment for individual patients by combining the demographic and clinical information, biomarkers and medical imaging data (44–47). Existing developments in precision medicine (44, 48–50) demonstrate that complex health-related big data of high quality are necessary, including lifestyle, nutrition, genetics, and environmental factors besides clinical, para-clinical, imaging and immunological or neurobiological parameters, which have to be analyzed and integrated in diagnosis, treatment and monitoring processes. To obtain big data and capture the bigger picture of a given individual on the way to precision medicine, Fagherazzi et al. recommend the method of “deep digital phenotyping”, which is a combination of deep phenotyping by collecting biomedical data in the real world and digital phenotyping by collecting digital biomarkers (42, 44, 51–53).

In the patient´s perspective, a more transparent disease understanding can enable the patient to take a more active role in decision-making, following the concept of patient empowerment (54). Better understanding and involvement of patients in therapeutic decision making leads to better treatment adherence, which is associated with higher efficacy and lower healthcare costs (55). Ultimately, all patients would have the opportunity to query their own data interpreted in the context of the world’s largest reference cohort and the latest data on available therapeutic options (56).

In relation to MS, deciding which therapy to use in a particular patient requires careful analysis of the patient’s disease course for high-risk factors for early progression, consideration of the efficacy and safety profile for a potential therapy, and a patient’s lifestyle and expectations (57). This is the only way to improve the precision of management for each patient, to improve prognosis and to establish an evidence-based framework for predicting response to treatment and personalized monitoring of patients. Precision medicine for pwMS involves the classification of disease subtypes based on underlying biology, not just clinical phenotype, and the development of predictive models that incorporate the integration of clinical, biological and molecular as well as current and emerging imaging markers with an understanding of the impact of the disease on the lives of individual patients (58–63). A complex data set could be the base of the DTMS as part of a digital data cloud that tries to simulate the same or very similar characteristics in terms of health status, risk factors and disease development as the real-world MS patient (43, 45).

## What Are Digital Twins?

### Origin and Concept Of Digital Twins

The concept of a “twin strategy” was generated from NASA’s Apollo program, which build two real identical space vehicles. One was launched onto the air space, the other stayed on Earth to mirror the conditions of the launched one (64). The first mention of the term “digital twin” can be traced back to the year 2003 when Grieves mentioned it in the context of manufacturing (64–66). Initially, the space industry was primarily concerned with the topic of DT. In 2012, the NASA and the U.S. Air Force jointly published a paper about the DT, which stated the DT was the key technology for future vehicles. After that, the number of research studies on DT in aerospace has increased and the DT was introduced into more fields such as automotive, oil and gas as well as health care and medicine. Examples are online operation monitoring of process plants, traffic and logistics management, dynamic data assimilation enabled weather forecasting, real-time monitoring systems to detect leakages in oil and water pipelines, and remote control and maintenance of satellites or space-stations. For instance, Singapore is developing a digital copy of the entire city to monitor and improve utilities (67). In recent years, the DT has been described more and more as a promising technology and it is expected that DTs will develop very strongly in the coming years and will bring a revolution in several industry sectors with the desire for online monitoring, increasing flexibility and personalized services (64). The availability of cheap sensors and communication technologies and the phenomenal success of technologies such as machine learning (ML) and AI, new developments in computer hardware as well as cloud and edge computing will rapidly drive the development of the DT (66).

Grieves (65) originally defined the DT in three dimensions: a physical entity, a digital counterpart and a connection that ties the two parts together. In most definitions, the DT is considered as a virtual representation that interacts with the physical object throughout its lifecycle and provides intelligence for evaluation, optimization, prediction, etc. (68–72). For instance, in the industrial sector the DT is used as an in silico presentation of technical applications in order to optimize them through computer simulations (67, 73, 74). As these definitions focus on three dimensions (physical, virtual, connection of them), Tao et al. added the two further dimensions data and services. The newly proposed definition can fuse data from both the physical and virtual aspects using DT data for more comprehensive and accurate information capture (64). Kritzinger et al. divide DT into three subcategories, depending on the level of data integration (75). Rasheed et al. present an example of a state-of-the-art DT of an offshore oil platform. The DT is continuously updated with sensor data almost in real time. The sensor data can be supplemented with synthetic data from simulators that provide physical realism at high spatio-temporal resolution. The DT not only provides real-time information for more informed decision-making, but can also make predictions about how the plant will develop or behave in the future. In an ideal environment, a DT is indistinguishable from a physical object in both appearance and behavior, with the added benefit of being able to make predictions about the future. In fact, the DT also offers the possibility for people to physically interact with the object using an avatar (66).

Overall, it must be noted that the topic of DTs is of such variety and complicated that it is almost impossible to cover all aspects as it has been covered by several reviews (66, 76–88). Up to now, there are currently no common methods, standards or norms for the development of DTs. In order to exploit the potential of DTs, there are still many challenges to be taken (66, 67).

### Digital Twins in Health Care

Focusing on the possibilities of DTs, medicine and healthcare are the areas that are likely to benefit most from the concept of DTs (66). There are several reasons for this. First, the number of intelligent portable devices and the organized storage of big data of individuals and cohorts is increasing. Second, human and thus medical thinking will eventually reach the natural limits of speed, complexity and performance. For HCPs, the massive and constant increase of knowledge in healthcare (e.g., differentiated diagnostics, more personalized therapies, interaction risks, active ingredients) is almost impossible to cope within daily work. HCPs are limited by everyday circumstances such as tiredness, time pressure and emotions. Especially in hospitals HCPs are under cost and time pressure and cannot always make decisions based solely on medical factors. And third, there is an increasing need for personalized and targeted treatment. As a result, various tools that enable precision medicine and simulation of therapies as well as prognosis of disease progression will inevitably find their way into the everyday life of HCPs, as is the case for already established different (clinical) decision support systems (CDSS) (89). The integration of technology and medicine is thus the main driver for intelligent and networked health. In this context, the statistical modeling of big data poses a particular challenge. Classical methods that examine associations between individual variables and a diagnosis or a course of disease reach their limits with the large number of statistical tests required and are also unable to uncover complex interactions between several variables and modalities in real time. Statistical significance, until now the primary measure of group-based, mechanistic research, also loses significance when, due to large samples, even the smallest effects exceed the significance threshold and thus the connection between significance and (clinical) relevance fades. ML is the key to creating direct clinical benefit. ML involves algorithms that can learn to solve a specific task autonomously based on data. These algorithms do not need to be explicitly programmed and can thus generate novel solutions to complex problems and tasks. Although classical statistical methods are capable of both correlation discovery and prediction, ML methods are better suited for identifying patterns, constructing features, and making predictions from large, complex, and heterogeneous data because they are usable and generalizable across a variety of data types and allow analysis and interpretation across complex variables. ML methods thus complement and extend existing statistical methods and can be used in highly innovative areas such as omics, radio-diagnostics, drug discovery, and personalized treatment. Of course, ML methods also have their limitations. The success of a ML project depends on the number of observations, the number of features, the selection and parameterization of the features as well as the quality of the underlying data and the chosen algorithm for the model (90, 91). ML also represents a component of AI research and development. AI is a computer system that is able to integrate relevant information and make a rational and logical decision that leads to the best possible outcome.

ML is an important component of a modern DT in healthcare (92), that can be defined as a “virtual mirror of ourselves that allows us to simulate our personal medical history and state of health using data-driven analytical algorithms and theory-driven physical knowledge” (93) as well as to exploit the synergies resulting from their combination. That is, a DT uses the induction approach (statistical models that learn from data) and the deduction approach (mechanistic models that integrate multiscale knowledge and data) to provide accurate predictions of pathways to maintain or restore health (45). A DT consists of numerous dynamic and multidimensional parameters. Dynamic data means that the data from which the digital image of the patient is created are both historically available data and continuously updating and accumulating data from that person’s life, e.g., data on the medical condition, data on the person’s living environment, data on how a drug is tolerated or a therapy is accepted. The multidimensionality of the data arises from the many different sources from which the data come, such as monitoring data, data from the patient’s social milieu, data from sensors, or clinical data. The dynamic and multidimensional nature of the data collected also distinguishes DT from other classical approaches such as clinical decision support systems (CDSS). A CDSS is used to make recommendations for appropriate tests and procedures from historical electronic health record (EHR) data using diagnosis of a condition and analysis of symptoms to help HCPs make informed decisions. The recommendation is the main component of a CDSS, which can be recorded in medical documents or coded in software as algorithms and rules (94, 95). However, the DT is not just a pure data collection approach for recommendations; it also correlates these data with each other and uses algorithms to incorporate the data meaningfully and purposefully into a simulation process with defined clinical (and economic) goals (95). The ability to simulate and model medical devices as well as pharmaceutical treatments on the computer enables faster and more cost-effective development than under real conditions (45, 48), without any risk for patients: “Making mistakes on computer models instead of people” (96).

The use of DTs in medical care is still in its infancy. So far, only in a few areas of medicine, DTs were applied, such as oncology (97–99), geriatrics (100, 101), cardiology (45, 102–106), epidemic outbreaks (107), genomic medicine (48, 108), internal medicine (109, 110), orthopedics (111) and vascular medicine (112, 113). For example, Corral-Acero et al. present early steps of a DT in the field of cardiovascular medicine by describing the synergies between mechanistic and statistical models, the pillars of the DT (45). Topol describes “high-performance medicine” with the help of AI for HCPs in different disciplines like radiology, pathology, dermatology, ophthalmology, cardiology and gastroenterology (114) and gives an overview over selected reports of machine- and deep-learning algorithms to predict clinical outcomes and related parameters. Laaki et al. developed the prototype of a DT for real-time remote control of remote operations over mobile networks (81). Bruynseels et al. show how DTs are based on in-silico representations of an individual that dynamically reflect molecular status, physiological state and lifestyle over time (46).

Concrete implementations of digital twins can already be found for organs such as the heart, for example, by the French software company Dassault Systèmes (115) or by Siemens Healthineers in Germany (116). Siemens Healthineers has used data collected in a huge database of more than 250 million annotated images, reports and operational data. The AI-based DT model was trained to weave data about a heart’s electrical properties, physical characteristics and structure into a 3D image. The technology was tested on 100 digital heart twins from patients treated for heart failure in a six-year study. Preliminary results of the comparison between the actual outcome and the predictions the computer made after analyzing DT status seemed promising. French startup Sim&Cure developed a DT system that virtualizes a patient-based aneurysm and surrounding blood vessels. After a patient with aneurysm is prepared for surgery, a DT represented by a 3D model of the aneurysm and surrounding blood vessels is created by processing a 3D rotational angiography image. The personalized DT allows surgeons to perform simulations and helps them gain an accurate understanding of the interactive relationship between the implant and the aneurysm. In less than five minutes, numerous implants can be assessed to optimize the procedure. Preliminary studies have shown promising results (117).

## Concept of Digital Twins in the Management of Multiple Sclerosis

Our vision is generating and implementing DTs in management of MS in order to improve diagnosis, treatment and management strategies as well as patient participation and compliance. DTs are a revolutionary tool for an improved characterization and prediction of disease course and for deep clinical phenotyping of pwMS (118). In this regard, big data analysis *via* ML supports visualization of the DTMS at different stages of MS and enables further therapeutic decisions. There are no elaborated DTs yet, but there are starting points and perspectives. For instance, Walsh et al. use an unsupervised ML model to learn the relationships between covariates commonly used to characterize subjects and their disease progression in clinical trials in MS (118). Recently, a research group from Sofia University in Bulgaria performed a first exercise of simulation of DTs. Petrova-Antonova et al. developed a web-based DT platform for MS diagnosis and rehabilitation that consists of two components: a transactional application that automates tests for MS diagnosis and rehabilitation, and an analytic application that provides data aggregation, enrichment, analysis, and visualization that can be used in any instance of the transactional application to generate new knowledge and support decision making. However, the analytical application is currently undeveloped and subject to further research (119).

**Figure 1:**
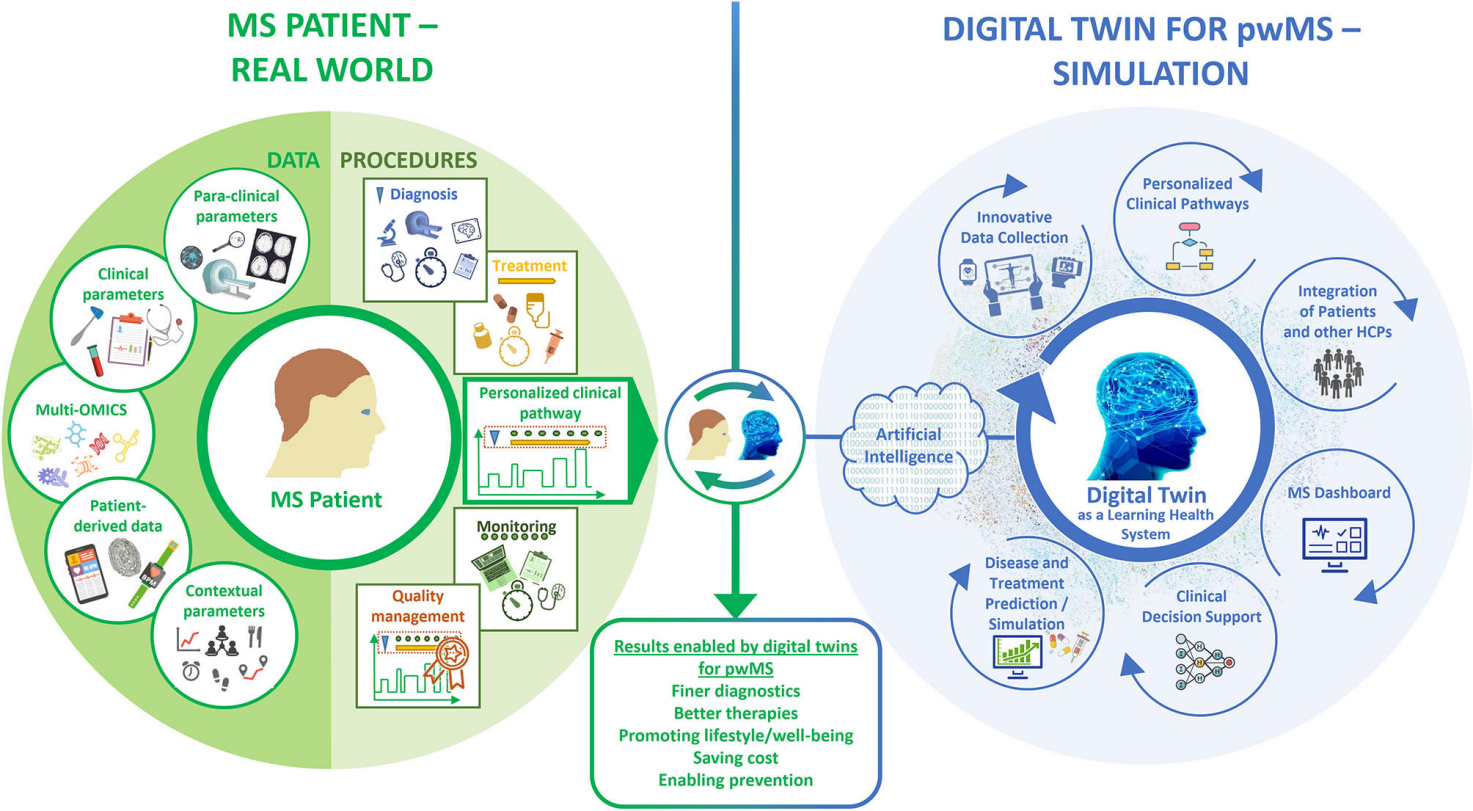
Concept of a digital twin for pwMS.

We consider that, due to the complexity and long-term nature of MS, a particularly large and multidimensional amount of data must be collected and organized for the construction of DTMS. These data must be of high quality, i.e. they must be collected correctly and represent the patient as accurately as possible. In addition to quality, a high quantity and frequency of data collection must also be achieved in the long term. To create DTMS and keep them updated with follow-up data, parameters related to the patients physiological status data (structured clinical data, para-clinical and multi-omics data, and patient-reported data) and to procedures (diagnostic workup, treatment, monitoring as integrated into personalized clinical pathways) should be collected, analyzed, visualized and correlated (**Figure 1**). The evolving and self-updating DTMS can be used simultaneously with ML algorithms to make smarter predictions and decisions as a learning health system (LHS) (120).

### Patients Physiological Status Data

Patients’ physiological status data content of DTMS includes structured clinical and para-clinical data, some of them as digital data, as well as multi-omics and patient reported data.

**Structured clinical data** are key parameters of deep clinical phenotyping and prerequisite for the data content of DTMS (30, 121). Taking the patient’s history is traditionally the first important step in the evaluation of pwMS, which focuses on relapses and/or disease progression in the different neurological functional systems. Contextual parameters including lifestyle factors, comorbidities (122), psychological factors, emotions and sociodemographic factors (123–125) must also be recorded, assessed through the medical record and the conversation between physician and patient. There are attempts to standardize and quantitate MS relevant neurological history, such as e.g. the MSProDiscuss tool in the assessment of secondary disease progression (126). Further clinical evaluation e.g. by neurological examination is indispensable in MS for the quantitative measurement of the extent of the disorder, which is in turn required to find out how the disease is evolving and the influence the different forms of treatment are having on it. In recent years, the Expanded Disease Disability Scale (EDSS) has been an essential, irreplaceable scale in MS which has been improved in the past years by different approaches (127–129). However, other additional clinical instruments have been introduced to quantitate the different multidimensional aspects of MS as fatigue, cognition or walking function (130, 131). The Multiple Sclerosis Functional Composite (MSFC) provides a functional assessment of different key functions (upper and lower extremities, cognition) that is used more and more frequently in MS and has been proven to be highly sensitive in the evaluation of very important clinical trials. These complex data could allow clinical phenotyping of MS in terms of disease activity (132) or symptom-specific phenotypes (133). Because DTs are data-driven approaches, it is not advisable to assume that the same monitoring procedures already used by the clinician in everyday practice are sufficient to establish a model for comprehensive digital representation of pwMS. Therefore, a combination of different clinical outcome measures is highly recommended (134). Initiatives to standardize the collection of clinical data are on the way (135).

**Para-clinical data** are of great importance for diagnosing, phenotyping and monitoring MS. Lab data ranging from standard laboratory to state of the art immunological or neurobiological parameters (136–139). Implementing standard lab data from clinical practice into a comprehensive approach of DTMS can complete the fundamental quest of real world evidence for individually improved treatment decisions and balanced therapeutic risk assessment (140, 141). As the MS disease process takes place in the CNS, analysis of cerebral spinal fluid is of high importance (123, 124). In addition to emerging immunological and neurobiological biomarkers, new technologies could be used for data collection for the DTMS as it has been described by Meyer zu Hörste (136) and will be described among multi-omics approaches. Neuronal destruction makers (e.g. neurofilament light chain) seem to be an excellent tool to measure subclinical MS disease activity in research and clinical studies (125, 142, 143), but final validation and transfer in clinical practice would be optimal in the setting of the multidimensional approach of DTMS.

The importance of CNS imaging has steadily increased in recent years and is expected to continue to grow in light of new sequencing techniques and applications related to pathophysiology and prediction (144, 145). As a biophysical technique for measuring magnetic properties and generating weighted images of relative tissue contrasts, MRI offers both volumetric and dynamic quantitative means of detecting pathological tissue changes. These represent a promising approach to optimizing MS management through *in vivo* monitoring in the assessment of the course of chronic diseases by recording their disease-related dynamics or treatment-induced effects (146, 147).

To implement imaging into DTMS, it is essential to standardize MRI acquisition (148, 149). The aim of this approach is to increase the sensitivity of MRI analysis to the smallest disease-related tissue changes. The acquisition of 3D-resolved sequences is important, as these, on the one hand, allow the free exploration of the image data by reformatting and, on the other hand, allow an optimal adaptation to the preliminary examination through modern 3D registration. In addition, only these 3D-resolved sequences form the basis for computer-assisted image data analysis and volumetric measurements, which should further increase precision in the future. Recent advances of CNS imaging could be probably transferred more easily into clinical practice by their integration into DTMS. Using this platform to put imaging data in context with other multidimensional data offers unique possibilities of validation and implementation. Thus, in future, quantitative MRI will enable a detailed characterization of brain tissue by generating a large number of numerical results (150). More than a thousand parameters can be generated if a detailed segmentation of the brain is considered, making group studies complex and inefficient by parametric techniques of data analysis (150). The large volume of MRI data can only be approached by AI, an essential tool of the DTMS (151). Finally, by measuring both volumetric and dynamic quantitative means (lesions and atrophy), different MRI phenotypes of individual patients can be described by MRI-categorization (152) which could be an important component of DTMS. In addition to MRI, data obtained through other imaging biomarkers such as OCT (153) or Positron emission tomography (154) can be used as well.

**Digital phenotyping**. Several clinical and para-clinical data can be collected digitally (digital phenotyping with digital biomarkers). Digital biomarkers are measures to collect objective data on biological (e.g., blood glucose, serum sodium), anatomical (e.g., mole size), or physiological (e.g., heart rate, blood pressure) parameter with the use of a biosensor (portable e.g. smartphones, wearable, and implantable devices), followed by the use of algorithms to transform these data into interpretable outcome measures (155–157). They are used for assessing e.g. cognitive function (158) or fatigue (159).

Sensor-based, portable measurement systems can be used both in the clinical setting and in the patients’ individual everyday life (at home). In the clinical setting functional tests and gait analysis can be performed digitally. The Multiple Sclerosis Performance Test (MSPT) is a digital adaptation of the MSFC with additional elements added (160, 161) and measures health status *via* iPad with questionnaire on health status, processing speed with Processing Speed Test (PST) (162), manual skills with 9-Hole-Peg-Test (9-HPT) and walking speed with Timed 25-Foot-Walk (T25-FW) (160). Multidimensional gait analysis can be performed with measurement of walking speed (T25-FW), measurement of endurance [2-Minute Walk Test, 2MWT (163, 164)] and measurement of balance and gait quality on a sensor-based walking mat (GAITRite^®^-System, Mobility Lab-System) (131). For the digital measurement of data in patient-specific everyday life (at home) there are various patient apps such as Floodlight, diverse fitness tracker and health apps available (165, 166). They make it possible to collect realistic data relevant to everyday life *via* remote sensing in addition to the regular medical consultations. Thus, a more comprehensive insight into the patients’ daily life as well as a more closely meshed progression monitoring is made possible. Clinical and para-clinical data (including lab and imaging data) are more and more collected in digital format and a standardized way which is an important step for integration in DTMS. A key role in the development of global standards of data related to patients or health cases is played by various organizations such as the Clinical Data Interchange Standards Consortium (CDISC), the Critical Path Institute (C-Path), and the Health Level 7 organizations (167). In clinical care, the development of digital neurological assessment tools such as Neurostatus-eEDSS and tablet-based MSPT, as well as real-time 3D motion capture systems for recording motor dysfunction in MS patients, play the most important role. The MS Data Alliance has already developed digital tools for aggregating, harmonizing, and sharing real-world data from multiple sources by creating a common data model. EHR also play a critical role in standardized and accurate digital documentation of clinical data, and several of these already exist, such as the MS BRIDGE, RC2NB, MSDS3D and MSBase EHR systems (168).

**Figure 2:**
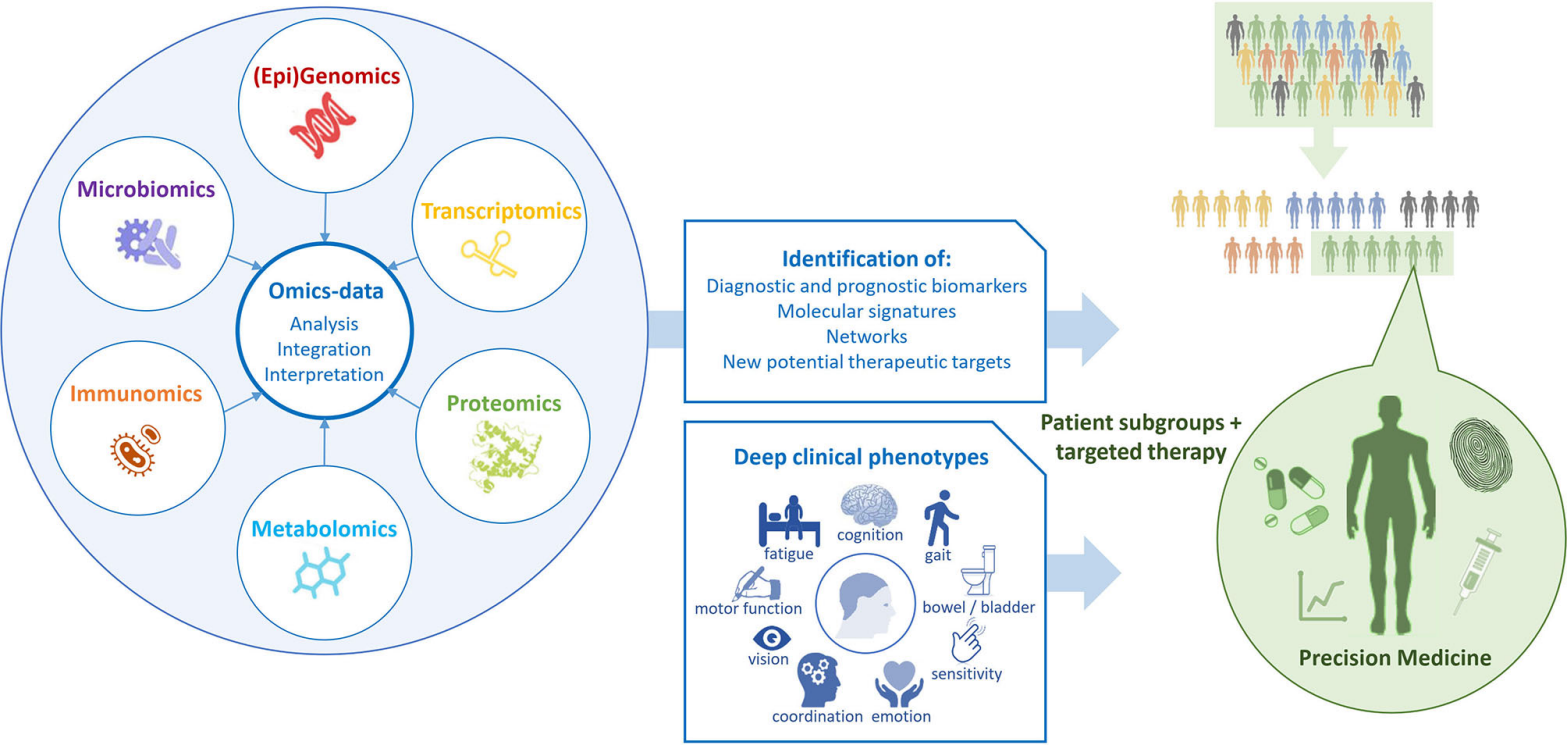
Multi-omics for precision medicine.

**Multi-omics** as innovative approach will have to be a part of the DTMS as well especially to increase knowledge about MS (169–171). The complex and dynamic processes in the neurobiological and immune networks are of significant importance in MS as in other chronic diseases. Advances in high-throughput “omics” technologies (e.g., genome, transcriptome, proteome, epigenome, metabolome) are enabling MS care to move from a “one-size-fits-all” toward a personalized approach analyzing the correlation of multi-omics with the clinical and para-clinical phenotypes of the individual MS patient (**Figure 2**). Multi-omics approaches involving large populations of pwMS and interrogating millions of markers with similar biochemical properties can help to elucidate the molecular mechanisms underlying MS and provide both potential biomarkers and pharmacological targets for a more detailed patient stratification and personalized treatments (172). Genomic and proteomic studies have sought to understand the molecular basis of MS and find biomarker candidates. Regarding genomic and proteomic studies, advances in next-generation sequencing and mass-spectrometry techniques have been of great importance to generate an unprecedented amount of relevant data (173). In order to study complex biological processes holistically, it is imperative to adopt an integrative approach. Multi-omics data should be combined to shed light on the interrelationships of the biomolecules involved and their functions. With the rinsing of high-throughput techniques and the availability of multi-omics data from a large number of samples, promising tools and methods for data integration and interpretation have been developed (174). In the field of MS, this strategy was successful for the development of novel data science techniques for exploring these large datasets to identify biologically relevant relationships and ultimately point towards useful biomarkers which have been discovered in recent years (124, 173).

**Patient-reported data** like questionnaire data complement the clinical data and complete the picture of the DTMS by including the patients’ perspective of their disease. They are divided into patient reported outcomes (PRO) and patient reported experiences (PRE). PRO is an umbrella term for health outcomes that are directly and subjectively reported by patients without interpretation of the patients’ response by a clinician or anyone else (175, 176). PRO are measured for outcomes like quality of life by the Quality of Life in Neurological Disorders (177, 178), and like walking and mobility skills by the Twelve Item MS Walking Scale (164, 179) or the Early Mobility Impairment Questionnaire (180). PRE measure “patient’s perception of their personal experience of the healthcare they have received” (181). PRE measures assess patients’ perception of their experience of the received healthcare collected through questionnaires (182). Efforts to standardize data are already underway. The PROMS (Patient Reported Outcomes for MS) initiative aims to identify PROs, including actively and passively delivered digital performance measures, to standardize outcomes in both research and clinical decision making (183).

Thus, model building for a DTMS already requires a comprehensive set of monitoring tools to be tested on a representative sample. To a certain extent, this also describes the scope of the instruments, which must later be applied to individual patients in practice in order to derive a comparable trajectories.

### Procedures

An optimal management of pwMS requires the performance of certain procedures as e.g. assessments of clinical and para-clinical parameters at high quality and at defined time points. In addition to the more general and non-concrete guidelines related to standard clinical practice, the Brain Health Initiative has provided for the first time specific “core,” “achievable,” and “aspirational” time frames for individual treatment steps in diagnosis, treatment, and monitoring (14). Achieving these standards of MS management in the individual patient to increase quality of care for pwMS will be facilitated by integrating such procedural components of these clinical pathways into the DTMS.

**Diagnosis** of MS is based on defined diagnostic criteria [McDonald criteria (5)] and relies on various examination methods (184), none of which alone is capable of making the diagnosis of MS as the differential diagnosis is quite complex. The procedural component of diagnostic workup in DTMS will assist in collecting data in optimal time considering type and stage of disease, pertinent symptoms and comorbidities, time between the first referral to the neurologist and MRI, etc.

**Treatment.** The therapeutic management in MS includes DMTs, treatment of acute relapses, and symptomatic therapies, which are usually combined and individually adapted. In particular, the history and the stage of the disease, degree of disability, the primary symptomatology, form and dynamics of the course of the disease, age, gender and desire to have children, concomitant and previous diseases, concomitant and pre-medication as well as the individual life situation of the patient must be taken into account. The DTMS will assist in the selection and monitoring of individual treatments. In order to assess possible adverse events and reactions, individual treatments need a defined treatment-related clinical pathway including clinical and para-clinical assessments, which have to be integrated into the DTMS.

**Monitoring.** An optimal primary goal of MS therapy should be the achievement of no evidence of disease activity (NEDA) (9, 185). Specifically, this means the absence of relapses, new or enlarged lesions on MRI, clinical disability progression and loss of brain volume (=NEDA-4). The NEDA status has to be assessed by procedures of MS monitoring to detect disease progression and relapse as well as the monitoring of disease activity and symptoms. The importance of frequent high quality monitoring in routine clinical management of MS is pointed out by numerous authors with reference to various studies and the comprehensive data on the significance of relapses, early EDSS changes, and the role of MRI (186–188). As monitoring of MS is a lifelong challenge for patients and HCPs, its integration into DTMS will assist in keeping up this essential long term assessment.

**Personalized clinical pathways** that integrate these procedures are also included in the design of DTMS and should be available for the HCP and patient together to ensure the best possible outcome.

### Construction of Digital Twins for Multiple Sclerosis

Prediction models based on statistical models already exist. For example, Stühler et al. and Kalincik et al. have investigated the individual response of pwMS to disease-modifying therapies using generalized linear models. However, in both studies, data density and quality were insufficient because, among other reasons, the cohorts were too small or there were data gaps in MRI data or data could not be comprehensively included (189–191). With the DTMS, all historically and currently available data should be continuously included in the analysis, if possible, to increase predictive power. In addition to the standardized and digitized parameters on patients’ physiological status data and procedures, the available prior knowledge in the field of MS should also be included in the construction of the DTMS. In addition to existing guidelines (14), this also includes further expert knowledge from the practice of clinical care of pwMS as well as possible knowledge about factors that can positively or negatively influence the disease, e.g. comorbidities, nutrition, physical activity and cessation of smoking.

Before the DTMS is implemented in practice, it is essential to check which data are absolutely necessary to collect and how the data collection can be done in such a way that it burdens the patient and HCP as little as possible. This is also important from an economic point of view, as the collection of all the above-mentioned data types is associated with high costs. This examination could be done by different tools. Basically, a targeted literature review on parameters particularly frequently used for prognostic purposes would be necessary, which could be complemented by a survey among experts. Since the strength of ML methods lies in discovering hidden patterns, test runs of the DTMS with the integration of different parameters (classes) would be conceivable, the results of which would be tested in a representative sample. Some work already provides clues in this regard. As Pinto et al. have pointed out in their work on prediction of MS progression using ML methods, relevant clinical information may include EDSS, functional systems and CNS functions affected during relapses, as well as age and gender (192). In any case, data acquisition should be done digitally and in an automated manner, if at all possible, with a view to minimizing patient disruption. There is a need for further research in this area which data have been collected from patient and HCP.

## Use Cases in Care of Multiple Sclerosis

DTMS perform a new kind of deep phenotyping by processing all data and procedural content in its complexity with innovative tools. Taking into account all previously defined medical and contextual parameters, which are very closely interwoven with the patient and his identity, the DTMS provides decision templates based on calculated probabilities. HCPs, patients, and all those involved in their care, have therefore an individualized roadmap of which examinations, tests, and therapies to pursue in the near future. In this process, the DTMS controls and monitors the entire disease management process and can correct any deviations. Thus, the DTMS is also a tool for measuring the process quality of a treatment. This results in a number of application scenarios that will fundamentally improve management of MS (**Figure 1**).

### Innovative Data Collection

For linking large amounts of data from different sources, suitable interfaces and modular database systems should be available that can integrate different external systems. The ability of different systems to work together is called interoperability. To achieve interoperability and also flexibility, the use of an interoperability standard, such as HL7 FHIR (193), and standard interfaces, e.g. IHE XDS.b for Germany (194), should be ensured (195). This is where a MS portal such as the Integrated Care Portal Multiple Sclerosis (IBMS) (195) could be used, to which both patients and HCPs can contribute different types of data. Patient data collected *via* apps or questionnaires flow into the patient portal, which is part of a management system for MS. The HCP, in turn, can see this data in the system and enter content related to the data and processes there. In the further course, data enter the database continuously, which can be used for the DT.

### Clinical Pathways

Clinical pathways are particularly suitable for the seamless care of chronically ill patients across various health sectors. They describe the entire path of patients during care (the “patient journey”) and unite the multidisciplinary setting, the local conditions and the current state of evidence research (195). Clinical pathways define goals and milestones of care and support shared decision making between HCPs and patients by also providing patients with a picture of their stage of disease (30, 195–198).

As intelligent systems, DTs traverse the clinical pathway, serving as a guide for HCPs and patients through treatment with an individual roadmap. Integrated into clinical management systems, clinical pathways can thus also serve as quality assurance tools for HCPs and patients. In this way, patients can actively participate in the quality improvement of their treatment process. HCPs, in turn, have the opportunity to optimize treatment steps based on specific quality indicators. These quality indicators are derived from existing MS guidelines and consensus standards [e.g., the International Brain Health Initiative consensus standards (14)]. On the one hand, they address temporal concerns for diagnosis, treatment, and monitoring phases, e.g., the maximum time between initial presentation and the acquisition of an MRI. On the other hand, quality instruments are integrated to measure the assurance of desired outcomes for pwMS, e.g., whether patients who have mobility or fatigue issues are offered support (199) or whether patients experience coordinated care with clear and accurate information exchange (200). Defining and measuring quality indicators is the goal of the currently running project “Path-based Quality Management in MS Care” (QPATH4MS) at the MS Center Dresden (Germany).

### MS Dashboard for Visualization

Visualization helps to present complex data in an understandable and clear way. The so-called MS dashboard visualizes high-dimensional disease characteristics and individual clinical pathways. The HCP can present the possibilities played out by means of the DT to the patient to discuss therapy options and clinical pathways with the patient. Through an adaptive display, it is possible to present the individual patient pathway, therapy options, treatment alternatives and the associated risks and challenges in a simplified form for the patient as a layperson and for the HCP as an expert. Within this framework, HCPs and patients can determine the ideal therapy and management of MS through shared decision-making. Thanks to the visualized simulation of the DT, the HCP has time to address all patients’ questions and concerns in detail. Examples of existing dashboards for displaying individual patient data at a glance include the walking assessment dashboard as part of the multidimensional digital patient management system MSDS^3D^ (201, 202), showing the results of clinical multidimensional walking assessment and daily smart monitoring longitudinally (131), and the MS BioScreen, that integrates multiple dimensions of disease information: clinical evolution, therapeutic interventions, brain, eye, and spinal cord imaging, environmental exposures, genomics, and biomarker data (56, 203).

### Integration of Patients and Other Healthcare Professionals

The visualization of the complex data involved in the medical and therapeutic decisions may foster the communication between HCPs and patients. This would support the involvement of patients in healthcare decisions and management of their disease. In this way, DTs also serve as a shared decision-making tool for HCPs and patients, who will play a much more active role in their own healthcare management in the future. For example, this could empower the patient to become an active member of the MS management team, from providing data (including data from biosensors, for example) to recording/tracking notable events and daily care to prognostic tools. As a result, a much more granular, continuous perspective on MS and its progression is provided, which would be more complete than traditional (brief and irregular) clinical assessments.

### Clinical Decision Support System

A DT also acts as clinical decision support system (CDSS) that supports HCPs in clinical decision making by providing evidence-based medical knowledge and patient-related information (204, 205). The goal is to enable the HCP to make the best possible clinical decision for the patient, with the best possible chance of a positive outcome. CDSSs are often supported by ML-based algorithms. The ambiguous patterns of MS (e.g., in etiology, progression, clinical presentation, and response to drug therapies) make ML algorithms optimal tools to automate the detection of patterns and regularities in MS data. CDSSs are very beneficial in the context of MS, but are not yet well established. There is an increasing need for CDSSs in MS to help HCPs make the right decision among multiple alternatives in time (206).

### Simulation and Prediction of Disease and Treatment Outcomes

Modeling the course of MS, especially predicting progression, is challenging due to the complexity of the outcomes and its varying course. The DT offers the possibility of predicting several probable disease courses and provides models for estimating possible treatment effects for individual patients. Taking into consideration all of a patient’s individual parameters, potential side effects, costs incurred, and individual circumstances and patient satisfaction, the DT can suggest the option with the highest benefit for the patient. There are initial approaches to predicting disease progression using ML. For instance, Pinto et al. used clinical information to develop a ML system to explore the disease evolution in pwMS in terms of conversion from RRMS to SPMS. EDSS score, majority of functional systems, affected functions during relapses, and age at onset were described as the most predictive features (192). Zhao et al. found that support vector machines incorporating short-term clinical and brain MRI data were better at predicting disease progression of MS and selecting patients for more aggressive treatments than logistic regression methods (207). Later, Zhao et al. compared common ML algorithms and so-called ensemble learning approaches. The latter were more effective and robust compared with single algorithms and offered increased accuracy for predicting disease progression of MS. Of the variables evaluated, EDSS, pyramidal function, and ambulation index were the most common predictors in predicting MS disease progression (208). Another study suggested that the concentration of serum cytokines could be used as prognostic marker for the prediction of MS (209). Data-driven subtyping and staging of MS could better predict subsequent clinical course and response to treatment compared with clinical classification or baseline EDSS. Data-driven subtyping has the potential to prospectively improve patient outcomes.

DTs help to understand disease’s dynamics and thus, advise HCPs on medication intake. With regard to drugs, it is quite conceivable that in the future clinical trials will also be conducted only with the help of DTs and no longer with the patients themselves.

From all that is known so far, the DT is a Learning Health System (LHS). LHS fuse healthcare delivery with research, data science, and quality improvement processes. The LHS cycle begins and ends with HCP-patient interactions and strives for continuous improvement in healthcare quality, outcomes, and efficiency (210). Based on the constantly new data collected through continuous monitoring and provided by the patient from the real world, the DT generates new knowledge, which in turn flows into the patient’s further treatment, which is thus continuously improved. The parameter data continuously flow into the calculations of the DT – with each piece of information, the phenotype can be described more precisely. The therapy can thus be continuously adapted to the patient’s disease state and life circumstances.

## Challenges of Digital Twins in Health Care

The use of DTMS promises to improve clinical decision making for individual patients, enhance patient communication, and improve quality of care. However, no uniform methods, standards, or norms yet exist for the development of DTs, and many challenges remain to unleash the potential of DTs (49, 66).

### Data Quality, Data Management and Algorithm Design

Poor or missing data and information can lead to improper models and incorrect recommendations (trash in, trash out). In order for the DT to be statistically indistinguishable from its real-world counterpart, the data on which the DT is based must be of high quality and represent the patient as completely as possible (118, 120). Data quality in the broader sense also includes the standardized collection or standardization of data to ensure their reliability and to enable longitudinal and cross-sectional comparisons of data. In this context, data should preferably be carried out in digital form or at least recorded digitally instead of in paper form in order to facilitate standardization and thus comparability. There is currently no generally accepted, standardized scheme for the collection, documentation, and evaluation of data in MS, although recommendations and guidelines from various expert groups exist, which have already been described in the sections on patients physiological status data and procedures (135). For the purpose of generating a sufficiently large amount of data describing pwMS in a standardized multidimensional manner, many years of multicenter data acquisition are required. Only on this basis is it possible to create the necessary “critical mass” of data in the required density to enable long-term estimation of therapeutic outcomes. In addition, multidimensional and unstructured large data sets must first be structured and then integrated into meaningful algorithms before meaningful models can be created (45, 95, 114). It should also be noted that the results of ML algorithms are usually based on a large number of parameters and criteria that can no longer be reproduced or fully understood by humans (135). Even if the models produce solid predictions, it may be impossible to deduce why they make good prediction.

### Data Privacy and Data Security

Before DTs are created, it is essential to clarify who owns which data at what point in time and for how long, who has access to it under which conditions and for how long, who actually owns the “end product” of the DT, and who can use it and under which conditions. It is imperative that suitable governance structures be created for this purpose. Furthermore, data security is very important to avoid data gaps that could potentially be used for hacker attacks to the detriment of patients. It is also necessary to ensure the protection of privacy, which becomes more and more difficult with the increasing functionality of techniques. Patients must also be confident that their data is secure, transparent and accessible to them. Otherwise, the collection of patient data could increase mistrust rather than confidence in health systems. Simply providing technological advances is not enough, it is also necessary to ensure that it serves to improve well-being. Therefore, data privacy and transparency of data use must be respected with the full consent of patients. Informed consent should explicitly state the purposes for which the data collected from patients will be used (49, 93, 120, 211).

### Ethical Concerns

DT models could exaggerate racial and other bias (46, 212) and could lead to or reinforce inequalities in health care (46): if a group is misrepresented in the data used to create models, this group may receive suboptimal treatment (213). An example shows that a computer model classifies patients with a history of asthma who have pneumonia as patients with a lower risk of mortality than those who have pneumonia only. However, the context was completely ignored, namely that this is an artifact of clinicians admitting and treating such asthma patients earlier and more aggressively (212). Another important ethical issue related to predicting the course of disease is whether and in what way the prognosis should be communicated to patients. How does a patient deal with the knowledge that, according to the prognosis, he or she will soon be in a wheelchair, for example? Do patients have a right to “not know”? In addition, the extent to which patients will be able to decide autonomously what is good or bad for them, and to what extent this will be determined by the algorithms that claim to propose the most optimal solution based on the available data, needs to be reconsidered. In this context, “dataism” could become a new form of medical paternalism. Patients must therefore develop an appropriate relationship with their personal DT and develop the ability to make informed decisions in the face of strong data-driven personalized models (46).

### Individual Concerns and Trust in Applications of AI

The role of humans or users of AI applications should not be underestimated, and trust is a crucial factor in this context (120, 214). The fear of new not-yet-established technologies like AI is a barrier to trust (120). HCPs may not trust the decisions of machines if they do not understand the involved algorithms. Additionally, HCPs could experience fear of being replaced by machines. However, AI will not replace the HCP (114), but will support and provide more time for consultation with the patient – one of the crucial aspects of medical care (215). Decisions based on AI can help the HCP make good decisions, if they “keep human intelligence up to date and take into account the social, clinical and personal context” (212). In the case that the DT’s recommendations contradict his or her own, the HCP must dispose of an action plan for further decision-taking. Otherwise, more data can contribute to the uncertainty of the medical thinking.

In order to establish the concept of the DT despite all the challenges mentioned, guidelines, gold standards, benchmark tests and governmental legislation, as has been achieved in Estonia, are therefore necessary (45, 114). Before using DTs in patient care, it is imperative that targeted studies, publication of results in peer-reviewed journals and clinical validation in a real-world environment are carried out (114). Nevertheless, HCP should proactively guide, supervise and monitor the introduction of DTs as partners in patient care (212).

## Discussion

**Figure 3:**
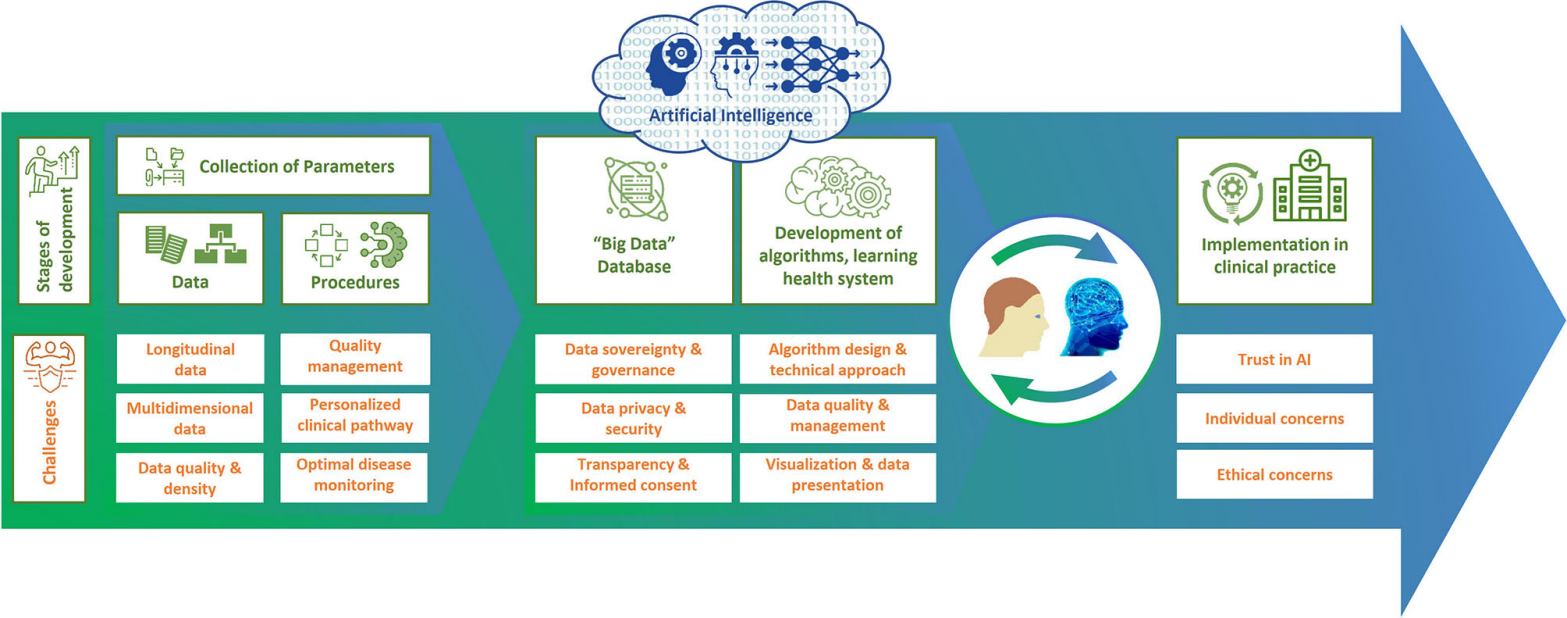
Time flow for digital twins.

With the development of a DTMS, it is possible to improve clinical decision-making for individual patients, patient communication, shared decision-making, and thus quality of care. Before DTs can be used in patient care, they must be validated by studies and experts, as well as by real-world investigations to show the effectiveness and safety of their methods. In addition, there are still a number of challenges to overcome on the road to using DTs, such as ensuring data security and privacy and the accuracy of the data on which the DT is based (**Figure 3**). It should also not be underestimated that the development of a DTMS is very complex and therefore expensive and may also increase the complexity of monitoring in clinical practice. Therefore, further research should be included in the development to inform which data contribute most to predictability, how this predictability can be assessed, and how this approach can be feasibly and cost-effectively integrated into health care. Further work will also be required to see whether and how predictive models can be constructed. However, a basic DTMS can serve as a starting point that will grow and evolve over time. During this process, the HCP should proactively guide, oversee, and monitor the introduction of DTMS as partners in patient care. By analyzing all possible factors of MS, DTMS will help make precision medicine and patient-centered care a reality in everyday life. This will ultimately refine diagnostics and monitoring, improve therapies and patient well-being, save economic costs, enable prevention, expand treatment options and empower patients.

## Author Contributions

IV and TZ designed and conceptualized paper as well as revised the manuscript for intellectual content. HI, AD, RH and AD revised the manuscript for intellectual content. All authors contributed to the article and approved the submitted version.

## Conflict of Interest

The authors declare that the research was conducted in the absence of any commercial or financial relationships that could be construed as a potential conflict of interest.
